# Association Between Quality of Life and Body Mass Index Among First-Year Medical Students in a Peruvian Private University: A Single-Center Cross-Sectional Pilot Study

**DOI:** 10.7759/cureus.81378

**Published:** 2025-03-28

**Authors:** María Mosqueira-Villegas, Victor Hugo Bardales-Zuta, Lissett Jeanette Fernández-Rodríguez

**Affiliations:** 1 School of Medicine, Universidad Privada Antenor Orrego, Trujillo, PER; 2 Department of Internal Medicine, Hospital I de Florencia de Mora EsSalud, Trujillo, PER; 3 Department of Radio-Oncology, Policlínico El Porvenir - EsSalud, Trujillo, PER

**Keywords:** body mass index, nutrition, obesity paradox, peru, quality of life

## Abstract

Objectives

Given that many university students engage in unhealthy lifestyle choices that can lead to overweight and obesity and that the same students often have poor quality of life (QoL) metrics, we aimed to search for a relationship between QoL and body mass index (BMI) among first-year medical students enrolled in a Peruvian university.

Materials and methods

This was a cross-sectional convenience sample study. It consisted of 150 first-year medical students who were generally healthy. The BMI and QoL of the participants were measured using a health scale and the Short Form-36 (SF-36) QoL-validated questionnaire translated into Spanish.

Results

The participants consisted of 89 women and 61 men, with a median age of 19 years. Sixty percent of students were of normal weight, and 13% were obese. BMI weakly predicted SF-36 score, R^2^= 0.035, F(1,148) = 5.39, p = 0.022, β = -0.72, p = 0.022, α = 86.78, p < 0.001. This correlation was mostly driven by the physical functioning and general health aspects of QoL.

Conclusions

A small, but statistically significant, negative correlation exists between BMI and SF-36 scores among the study participants, indicating that obese first-year students had lower QoL than their normal-weight counterparts. This suggests that nutritional intervention among first-year students may improve QoL in subsequent years of their university career.

## Introduction

University student life is marked by irregular and demanding schedules with limited free time, stress and social pressure to perform, and financial constraints. These characteristics of student life often lead to unhealthy lifestyle choices, such as a poor diet of calorie-dense but unnutritious food and a sedentary lifestyle, which, in turn, lead to negative health consequences such as weight gain and poor self-reported quality of life (QoL), especially in the first and last years of study [1,2].

Obesity, defined as a body mass index (BMI) of more than 30, is a risk factor for both short- and long-term negative health outcomes, including diabetes, cancer, hypertension, and depression [3,4]. Of particular interest is that there is also a well-established but sometimes weak association between BMI and anxiety [5], mental health [6], work capacity, and QoL [7-18].

QoL is a multidimensional concept that includes several factors of a person's subjective perception of well-being. Different surveys have been proposed to measure QoL, including the Short Form-36 (SF-36), which measures eight different dimensions associated with QoL, which are physical functioning, limitations caused by physical problems (role physical), body pain, general health, vitality, social functioning, limitations caused by emotional problems (role emotional), and mental health. The SF-36 measures the mental and physical components of QoL separately, and although the original authors did not recommend combining them, several subsequent authors have attempted to do so to create an overall QoL score [12,19,20].

Given the long-term negative consequences of poor lifestyle choices among university students, understanding the link between weight gain, diet, and QoL can inform early intervention protocols. This is particularly important in medical students, who often report having a lower QoL than the general population [14,21,22]. In this study, we examine the association between self-reported QoL and BMI among first-year medical students attending a private university in Trujillo, Peru. Although previous work has already shown a negative association between BMI and QoL, we revisit the topic including only first-year medical students, where not only the stress of adjustment to a new academic routine is particularly pronounced but also intervention would possibly be more effective: making a positive difference for the rest of their student careers and beyond [12].

## Materials and methods

Study type

An observational and convenience sample cross-sectional study was carried out at the Medical School of Antenor Orrego Private University (FHM-UPAO). In total, 150 students from the campus in Trujillo, Peru, were included.

Ethical aspects

Ethical approval for the study was provided by the Bioethics Committee of the Antenor Orrego Private University with resolution number 01153-2024-UPAO before data collection began. Study recruitment and data collection took place in June 2024. One of the authors, who is a professor at FHM-UPAO, described the study in person to her class and invited the students present to voluntarily participate. All students who agreed to participate in the study provided their written informed consent before proceeding with the study. Data was depersonalized to protect confidentiality.

Sample size

The sample size was estimated using the sample size formula for a very large population, a margin of error of 0.08, a probability of 0.5, and 95% confidence. These figures indicate a sample size of 150 for the study.

Eligibility criteria

Only medical students attending FHM-UPAO who completed the first partial examination of the second semester of their first year were eligible for this study. Additionally, the participants must have completed the BMI measurement and the SF-36 questionnaire. The participants were excluded if they had chronic pathologies or if they did not complete the questionnaire or measurements. This allowed for the study to focus on physically healthy students and avoid biases associated with chronic disorders.

Data collection

Participant age, self-reported biological sex, body height, body mass, and SF-36 responses were recorded for this study. To determine body mass and height, a Greetmed GT131-200 (Ningbo, China) health scale was used. The participants were asked to remove outer clothing, shoes, and pocket items before being weighed and measured. Body height was measured with the participants standing straight with their backs and head against the stadiometer. The age and sex of the participants were self-reported.

QoL data was collected using a modified Spanish version of the SF-36 survey [20]. This questionnaire contained 36 questions where the participants are allowed to choose their responses from two or more choices. Each question was designed to address one of the eight dimensions of QoL that the test measures: physical functioning, limitations caused by physical problems (role physical), body pain, general health, vitality, social functioning, limitations caused by emotional problems (role emotional), and mental health. A copy of the questionnaire used is located in the Appendices. The responses to the question regarding changes in health were not used in the present analysis because this study was focused on present health perceptions, not changes in general health status. Furthermore, this question was not used in calculating the individual QoL dimensions. The participants usually completed the questionnaire in 10-12 minutes.

Measurement bias was addressed using the same instruments, techniques, and measurement technicians throughout the study. Instruments were calibrated and verified before measurements were taken. Selection criteria were designed to not exclude those who had differing BMI or QoL.

Quantitative variables

The BMI was calculated by dividing the body mass (kg) by the square of the body height (m). The numerical value of BMI was then used to calculate nutritional status using the following definitions: underweight, >18.5; normal weight, 18.5-24.9; overweight, 25.0-29.9; obesity grade I, 30.0-34.9; obesity grade II, 35.0-39.9; and obesity grade III, ≥40.

A modified SF-36 questionnaire was scored using a 0-100 scale for each question. To score a question, the responses were first ranked from the response corresponding to the lowest QoL to the highest QoL. The point values for each ordered response were assigned by dividing 100 by one less than the total number of responses and then multiplying that value by one less than the rank of the answer. Standard methods were then used to calculate scores for QoL dimensions and a total QoL score [20].

Statistical analysis

Shapiro-Wilk normality, Pearson's chi-square (χ²), Fisher's exact, ANOVA, Kruskal-Wallis H, and Wilcoxon Rank Sum tests were performed using R version 4.4.2 (R Foundation for Statistical Computing, Vienna, Austria). Cronbach's alpha, Mann-Whitney U test, Pearson's correlation, and ANOVA tests were performed using SPSS version 27 (IBM Corp., Armonk, NY). Effect size calculations were calculated using PSPP version 2.0.1 (Free Software Foundation, Boston, MA). Where possible, different programs were used to calculate the same tests to confirm their results. A probability of <0.05 was considered statistically significant.

## Results

Validation of the SF-36 survey

A pilot group of 50 students from FHM-UPAO was evaluated with the SF-36 test to measure the test's reliability in the study population. The results of this test obtained Cronbach's alpha of 0.762, indicating that the study population both understood the test and that the test results were reasonably reliable.

Study demographics

The participants consisted of 81 women and 69 men. Both mean and median age was 19: 72 students were 17 or 18 years of age, 62 students were 19 or 20 years of age, and 16 were between 21 and 30 years of age. The age distribution was skewed toward younger ages; the Shapiro-Wilk test showed a significant deviation from normality, W(150) = 0.66, p < 0.001. Given that only the participants who completed the SF-36 and anthropomorphic measurements were included, there were no participants with missing data.

Body mass index

**Figure 1:**
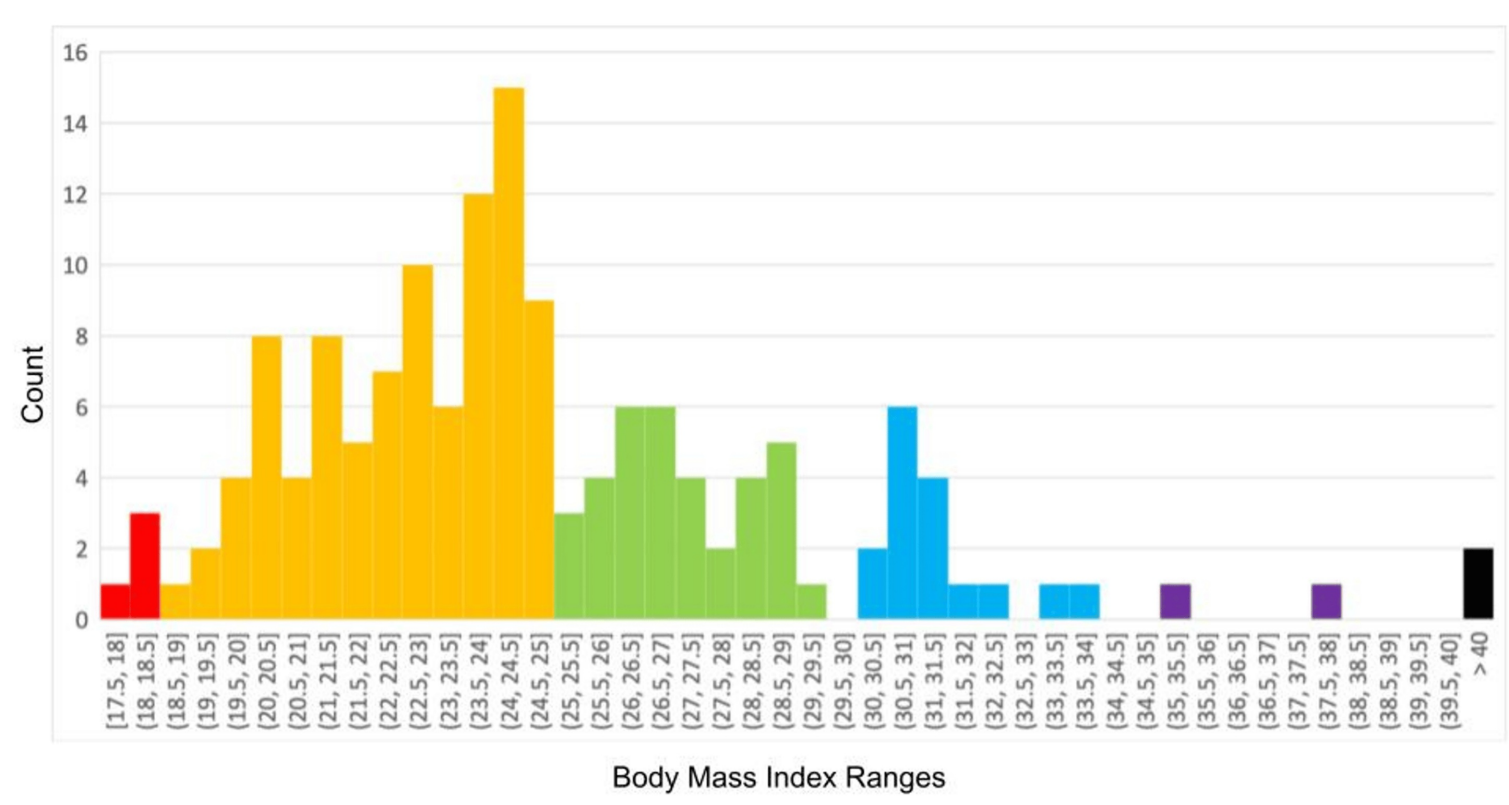
Histogram of the BMI for 150 medical students attending FHM-UPAO, showing a skew toward lower BMI and a range that spans all BMI classes. BMI classes are colored with underweight in red, normal weight in orange, overweight in green, and the three classes of obesity in light blue, violet, and black. BMI, body mass index; FHM-UPAO, Medical School of Antenor Orrego Private University

The BMI ranged from 17.8 to 41.3, spanning a range from underweight to type III obesity. The median BMI was 24.0, with the Shapiro-Wilk test showing a significant deviation from normality, W(150) = 0.93, p < 0.001 (Figure 1). Four students (3%) were underweight, 90 students (60%) were of normal weight, 36 (24%) students were overweight, and 20 students (13%) were obese. The underweight participants were all under 20 years of age: one was 17 years old, two were 18 years old, and one was 19 years old.

SF-36 test scores and BMI

**Figure 2:**
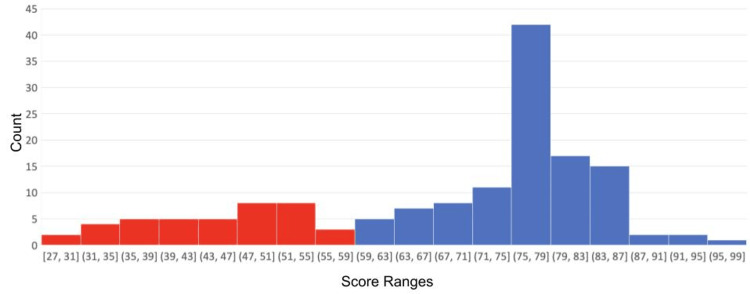
Histogram of SF-36 scores for 150 medical students attending FHM-UPAO. This bimodal distribution is divided into two groups: lower test scores (red) and higher test scores (blue). SF-36, Short Form-36; FHM-UPAO, Medical School of Antenor Orrego Private University

SF-36 test scores had a bimodal distribution with two peaks around 47 and 77 (Figure 2). The median QoL was 76.5, the mean QoL was 68.9, and the most common test score was 78.

The inspection of Figure 2 suggested a cutoff test score of 59 (59 and lower were low scores, while 60 and above were high scores), providing a group of 110 high scorers and a group of 40 low scorers. Of the high scorers, 35 (31.8%) were overweight or obese, while 21 (52.5%) of the low scorers were overweight or obese. Pearson's chi-squared test showed that there was a significant association between the test score and the proportion of overweight, χ² (degrees of freedom {df} = 1, N = 150) = 5.36, p = 0.021, OR = 2.37, effect size (Phi) = 0.20. Fisher's exact test for count data gave a similar result: p = 0.023 with an OR of 2.36 and a 95% confidence interval of 1.06-5.30. This association between the proportion of overweight or obese and test scores remained significant if the cutoff test score varied between 50 and 65.

**Figure 3:**
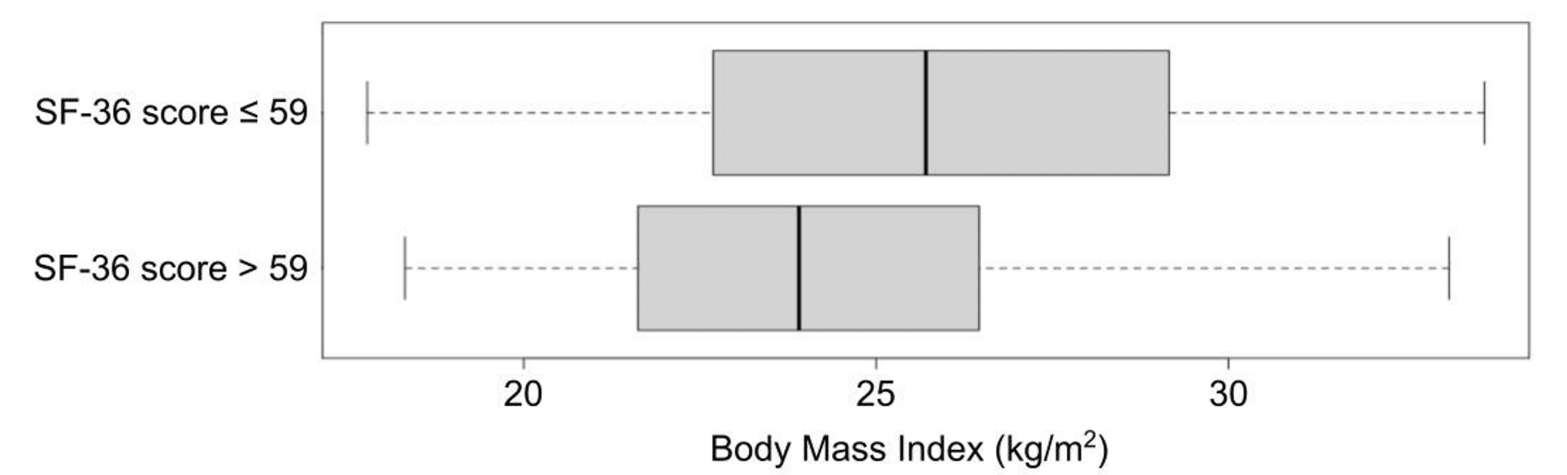
Box plots of BMI (horizontal axis) versus low (top) and high (bottom) SF-36 scores. BMI, body mass index; SF-36, Short Form-36

BMI can also be directly compared between groups (Figure 3).

**Figure 4:**
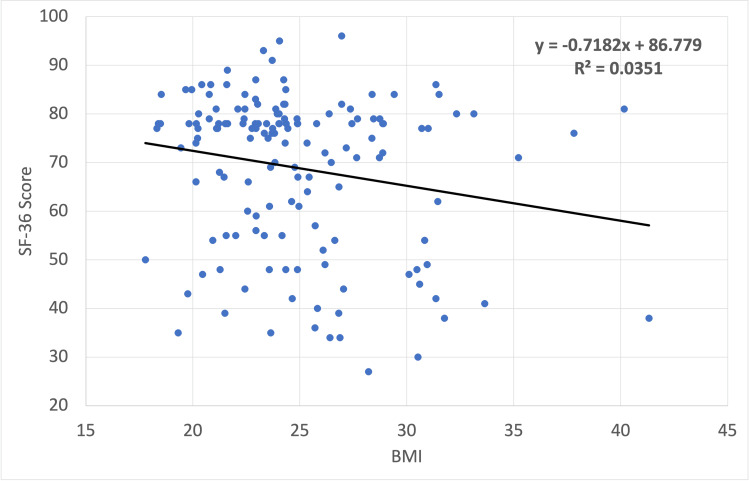
Plot of SF-36 score versus BMI for 150 medical students attending FHM-UPAO. Correlation analysis shows a weak negative correlation between SF-36 score and BMI (black line). SF-36, Short Form-36; BMI, body mass index; FHM-UPAO, Medical School of Antenor Orrego Private University

If the numerical values of BMI and SF-36 score are used directly, BMI weakly predicted SF-36 score, R^2 ^= 0.035, F(1,148) = 5.39, p = 0.022, β = -0.72, p = 0.022, α = 86.78, p < 0.001. This means that incremental increases in BMI are correlated with incremental decreases in the SF-36 test score (Figure 4).

Of the eight aspects of QoL measured by the SF-36, only physical functioning and general health significantly varied with BMI: BMI predicted physical functioning, R^2^ = 0.055, F(1,148) = 8.55, p = 0.004, β = -0.8, p = 0.004, α = 112.22, p < 0.001, and BMI predicted general health, R^2^ = 0.031, F(1,148) = 4.68, p = 0.032. β = -0.7, p = 0.032, α = 79.37, p < 0.001. A Bonferroni correction for the repeated correlation measurements excludes the general health aspect, while physical functioning and overall score remained significant. Other parameters, including those associated with the mental component of QoL, did not show a statistically significant correlation.

QoL across BMI groups

A different way of analyzing the data is to first group the participants by BMI, underweight or normal, overweight, or obese, and then compare SF-36 components. Table 1 compares these parameters, showing the average score for each of the eight components of QoL by BMI group.

**Table 1:** Mean SF-36 dimension score by BMI group. SF-36, Short Form-36; BMI, body mass index

	Physical Function	Role Physical	Body Pain	General Health	Vitality	Social Functioning	Role Emotional	Mental Health	Overall Score
Normal Weight or Underweight, n = 93	93	81	75	63	54	65	59	57	72
Overweight, n = 37	94	83	78	64	58	70	68	59	66
Obese, n = 20	83	66	80	53	54	53	35	49	60

A Kruskal-Wallis H test was completed for test scores sorted by BMI group for each of the eight QoL dimensions and overall score. Statistically significant differences were found for physical function, general health, social functioning, role emotional, and overall score between the three BMI groups. P-values ranged from 0.011 for overall score to 0.036 for social functioning. Given that nine repeated tests were performed on the data, the Bonferroni cutoff p-value would be 0.006, indicating that none of the parameters would reach statistical significance. A two-way ANOVA on the same data found that the differences in the averages within groups (BMI groups and QoL dimensions) were significant (p < 0.001), but the differences between the sample averages of all groups were not statistically significant. In other words, the effect of QoL components on the cells' means does not depend on the effect of the BMI group and vice versa (p = 0.187).

## Discussion

University students suffer disproportionately from poor dietary habits and poor QoL, as well as high levels of stress, especially in the first and last years of their program [2,14,21,22]. This study only included first-year students midway through their first years, offering a window into both BMI and QoL metrics of this population while it adapts to the stressors of university life. Furthermore, medical students generally do not have sufficient knowledge about nutrition, which can be improved with intervention [23,24]. Since students are generally young (median of 19 years in this study), unhealthy habits reinforced during the university career will have a more strongly negative impact over time than habits developed later in life, highlighting the need for early nutritional intervention. The proportion of obesity in the study population (13%) aligned closely with a study conducted on the Peruvian population when adjusted for age, indicating that there is little difference between the general population and medical students in terms of obesity determined by BMI [25].

The results analyzed here demonstrate that there is a weak but statistically significant negative correlation between BMI and SF-36 test score, especially in the physical aspects of QoL, although some other weak correlations may be present when some dimension scores are compared by the BMI group. This data supports an international trend that obesity negatively affects QoL and vice versa among students and the population in general [10,26]. Studies among university students around the world also support this consensus, with QoL and good nutritional status positively correlated. For instance, a study among Turkish university students found that physical functioning and the role physical aspects of QoL vary inversely with BMI, but the mental component does not [13]. Likewise, a study of Chilean adolescents showed that overweight, obesity, and low academic performance were associated with lower health-related QoL measured by a different test [16]. Another study from the same country corroborated the negative relationship between BMI and many aspects of QoL [14]. Studies from Singapore [8], Canada [7], Iran [18], the United States [21], and the United Kingdom [9] also showed a negative relationship between BMI and QoL, especially in the physical dimension.

However, a study of medical students conducted in Lima failed to show a statistically significant association between BMI and the eight aspects of QoL, but the direction of the association was the same [12]. The proportion of obese participants was also lower than in this study, which could have explained the lack of significance. A study from Indonesia found that the mental aspects of QoL decreased with increasing BMI, while the physical aspects of QoL did not have a significant correlation, although the direction of the correlation was negative [17]. Similarly, a study from Romania failed to find a statistically significant correlation for overall QoL, but there was a relationship between BMI and specific aspects of QoL [15]. In a Chinese study, the mental components of QoL seemed to improve for mildly obese people, but increasing BMI caused a downward trend in physical functioning [11]. These results, taken together, suggest that a negative correlation between overweight and QoL likely exists but is weak and can often be masked by other important factors that cause overweight and/or different QoL outcomes.

Only a small proportion of the variation in BMI can be explained by QoL and vice versa, so other factors are involved in these results. There are several potential confounding variables present in studies that measure weight and well-being among university students. One of them is physical activity, which has its own relationship with QoL and BMI [27]. Furthermore, different aspects of student life, such as whether a student lives in a dormitory or with family or has access to financial resources or healthy food, can act as confounders [13]. Also, the self-perception of weight status has an impact on health-related QoL [28]. Understanding the causes of obesity is also complex as genetics, pharmaceuticals, microorganisms, and maternal factors have been shown to be involved, so it is difficult to determine to what extent QoL plays a role in obesogenesis [3,4]. Interestingly, factors that can affect QoL are also listed as causes of obesity, such as stress, sleep quality, and socio-environmental factors [3,4]. Further compounding the complex relationship between QoL and BMI is that the correlation is probably not linear, as underweight people also report lower QoL parameters [9,11]. Also, there are different methods to define QoL and obesity, such as different surveys, waist-hip circumference, and body fat percentage, which make cross-comparison difficult.

Although this study sheds light on aspects of QoL and BMI, it does have limitations. Given that it is a transversal study, it cannot establish a cause-and-effect relationship between QoL and BMI or vice versa. Also, this study did not follow students over time to see how BMI and QoL aspects evolved during the participants' university careers. Although BMI is a common way to asses obesity, other indicators such as waist circumference, waist-to-hip ratio, and body fat percentage could have also been used to strengthen the study. Furthermore, the study was conducted at a single center with a small number of participants, so the conclusions may not be generalizable to other populations or universities. For this reason, larger population samples from multiple universities observed over time would provide more definitive information. Also, this study did not record information on diet quality and exercise among the participants to establish whether or not a healthy lifestyle was followed. Sample selection bias may also have been present, as there was no ability to determine whether nonparticipating students were systematically different than the participants due to ethical reasons and non-probabilistic sampling.

## Conclusions

Two important conclusions can be determined from these results: (1) It is likely that both underweight and obese people have lower reported physical dimensions of QoL, and (2) normal-weight and slightly overweight people tend to have higher QoL scores. It remains to be determined exactly where the horizontal asymptote of this curve is. This negative association between high BMI and QoL is somewhat weak but frequently statistically significant, including in the population studied here.

These results suggest that interventions to improve eating and lifestyle habits among medical students can also improve the physical aspects of their QoL, which is usually lower than the general population. Further directions for study may include looking for gender-specific relationships, expanding the study to compare students from other years of medical school, and evaluating whether nutrition education can improve QoL scores.

## Appendices

Data availability statement

The datasets generated during and/or analyzed during the current study, as well as an example data collection sheet, SF-36 questionnaire, and informed consent form, are available in the Figshare repository (https://www.doi.org/10.6084/m9.figshare.28012679).
